# Identification of Phenolic Compounds Present in Three Speedwell (*Veronica* L.) Species and Their Antioxidant Potential

**DOI:** 10.3390/antiox13060738

**Published:** 2024-06-17

**Authors:** Ivana Vrca, Stjepan Orhanović, Ivana Pezelj, Karolina Sušić, Valerija Dunkić, Dario Kremer, Marija Nazlić

**Affiliations:** 1 Faculty of Science, University of Split, Ruđera Boškovića 33, 21000 Split, Croatia; ivrca@pmfst.hr (I.V.); stipe@pmfst.hr (S.O.); izaper@pmfst.hr (I.P.); ksusic@pmfst.hr (K.S.); dunkic@pmfst.hr (V.D.); 2 Faculty of Agriculture, University of Zagreb, Svetošimunska cesta 25, 10000 Zagreb, Croatia; dkremer@agr.hr

**Keywords:** *Veronica*, polyphenols, LC-MS/MS technique, antioxidant activity, DPPH, ORAC

## Abstract

Extracts from *Veronica* species (speedwells) are known for the various biological activities they show, such as cytotoxic, antimicrobial, anti-inflammatory, and antioxidant activities. Also, the plants from this genus are known as medicinal plants used in traditional medicine worldwide. Phenolic compounds are specialized metabolites that contribute to biological activity the most. Therefore, the aim of this research is identification and quantification of phenolic compounds present in three *Veronica* species (*Veronica anagallis-aquatica* L., *Veronica persica* Poir., and *Veronica polita* Fr.) using the liquid chromatography-mass spectrometry (LC-MS/MS) technique. All extracts were tested for antioxidant activity with two methods: DPPH (2,2-diphenyl-1-picrylhydrazyl) and ORAC (oxygen radical absorbance capacity). Also, standards for compounds that were detected in the highest amount in all species were also tested for antioxidant activity. Three different solvents (pure methanol, 80% ethanol, and water) were used for the extraction of phenolic components and their comparison in order to test their antioxidant activity as a final goal. The main compounds present in the tested *Veronica* extracts were: *p*-hydroxybenzoic acid, vanillic acid, caffeic acid, gentisic acid, and apigenin. *V. anagallis*-*aquatica* contained the highest amount of phenolic components in comparison with the two other tested species, *V. persica* and *V. polita*. Caffeic acid showed the highest antioxidant activity in both studied methods with an IC_50_ value for DPPH activity of 1.99 µg/mL. For the plant extracts, in general, methanolic/ethanolic extracts showed higher activity than water extracts in both methods which was expected, as organic solutions extract more phenolic compounds. This research points to the potential application of extracts of different *Veronica* species for antioxidant activity.

## 1. Introduction

Plants are known for their diverse biological activities and for their specific chemical identity that enables them to synthesize metabolites such as phenolic compounds, saponins, terpenes, coumarins, and alkaloids, which possess protective properties against abiotic and biotic stresses [1]. The species of the genus *Veronica* L. (speedwells) are known for their traditional uses and the various biological activities they exhibit [2]. Some of the species have been used to treat influenza, respiratory diseases, haemoptysis, laryngopharyngitis, cough, hernia, and cancer; as diuretics; and in wound healing. For example, *Veronica officinalis* L., *V. beccabunga* L., and *V. spicata* have been used in the traditional medicine of Balcanic people for treating various conditions such as liver, spleen, kidney, and urinary bladder diseases, in the healing of snake stings, wounds, skin eruption, eczema, and ulcers, and as expectorants for cough and throat rinsing [3]. The genus *Veronica* is extremely variable in its morphology and its species are very well adapted to different living conditions. With around 450 species, it is the largest within the order Lamiales (family Plantaginaceae) [2]. Most representatives of this genus grow in areas with a Mediterranean climate [4]. So far, only a few studies have confirmed that certain species have biological activity [5]. Recent pharmacological research and clinical practice have shown that extracts or monomeric compounds from *Veronica* species have several pharmacological properties, such as anticancer, antibacterial, anti-inflammatory, antiangiogenic, antioxidant, antineurodegenerative, neuroprotective, and hepatoprotective effects both in vivo and in vitro [6]. There are few studies regarding the pharmacological activities of these three chosen species. For example, in one study a methanolic extract of *V. anagallis-aquatica* was tested against five bacterial strains and two yeast strains and these extracts showed significant inhibition compared to gentamicine. Methanolic extracts of *V. persica* showed good antifungal activity against *Candida albicans* and *Aspergillus niger*. For the species *V. persica* and *V. polita*, cytotoxic activity was studied for their methanolic and water extracts and they demonstrated potent activity against mouse melanoma cells [7]. Previous studies of secondary metabolites isolated from many species of the genus *Veronica* have included free volatile compounds in the form of essential oils and hydrosols [8,9,10,11,12,13] and bound volatile compounds such as iridoids, saponins, and phenolic components [2,4,6,14,15,16,17,18,19,20]. For the species *V. persica*, different iridoid glycosides were isolated and identified, such as aucubine, catalposide, amphicoside, veronicoside, and others. For the species *V. persica*, some of the identified phytochemical constituents were also persicoside, acteoside, isoacteoside, calendin, and tyrosol [7]. In another study, main iridoid constituents of the extract of *V. anagallis-aquatica* were veronicoside, catalposide, and verproside [21]. In the previous studies on phenolic compounds, the most abundant compounds for *V. persica* were luteolin, rutin, rosmarinic acid, *p*-hydroxybenzoic acid, protocatechuic acid, and 4,5-dicaffeoylquinic acid [22]. The study of phenolic compounds in plants is important due to their potent antioxidant properties, whose effect in preventing various diseases associated with oxidative stress is evident [23]. It is a known fact that excessive production of reactive oxygen species (ROS) is extremely dangerous for living organisms, damaging key cellular components such as DNA, proteins, and lipids [24]. Therefore, the search for new products that reduce the damage caused by free radicals is an extremely important research task in pharmaceutical research [24]. The extraction of phenolic compounds is the first and most important step in research, and they can be extracted from fresh, frozen, or dried plant material. The yield depends on the polarity of the solvent, the extraction time and temperature, and the ratio of sample to solvent. The most commonly used solvents for the extraction of bioactive components from medicinal and traditional plants are polar solvents (e.g., water and alcohols), medium-polarity solvents (e.g., acetone and dichloromethane) and non-polar solvents (e.g., n-hexane, ether, and chloroform) [25]. The solubility of phenolic compounds is determined by the chemical nature of the plant sample [23]. For this reason, it is important to investigate the influence of different solvents on the extraction of the desired bioactive components. Therefore, the aim of this research was to extract phenolic compounds with different solvents (pure methanol, 80% ethanol, and water) from three *Veronica* species (*V. anagallis-aquatica*, *V. persica*, and *V. polita*). The phenolic compounds were identified and quantified using the LC-MS/MS technique. Antioxidant activity was determined using two methods: DPPH (2,2-diphenyl-1-picrylhydrazyl) and ORAC (oxygen radical absorbance capacity).

## 2. Materials and Methods

### 2.1. Plant Preparation, Extraction and Identification of Phenolic Compounds 

**Figure 1:**
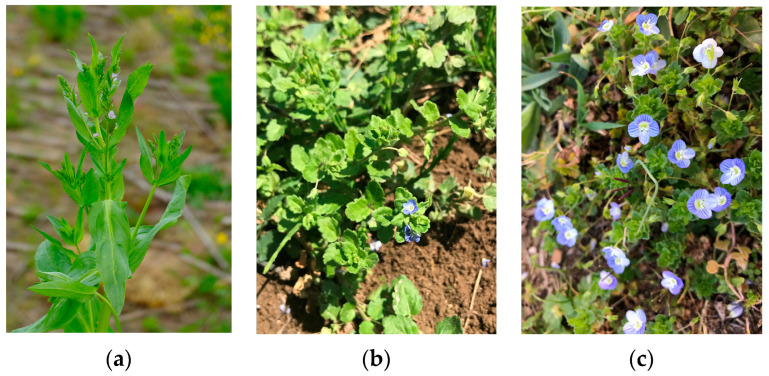
(**a**) *V. anagallis-aquatica* L. (author: Dario Kremer); (**b**) *V. polita* Fr. (author: Marija Nazlić); and (**c**) *V. persica* Poir. (author: Marija Nazlić).

Plant material (*Veronica anagallis-aquatica* L., *V. persica* Poir., and *V. polita* Fr.) was collected during the flowering period in May and June 2022 at various locations in Croatia (Table 1). The voucher specimens were deposited in the herbarium of the Laboratory of Botany (HPMF-HR) of the Faculty of Science, University of Split, Croatia (Figure 1a–c). 

All specimens were air dried in a single layer for ten days and protected from direct sunlight. Before the extraction of phenolic compounds present in different extracts of the above-mentioned *Veronica* species, the plant material was milled to a fine powder using a coffee grinding machine (Kaffeemühle, Kaufland Stiftung and Co., Neckarsulm, Germany). The powdered plant material (0.5 g) was extracted by maceration with 25 mL of three different solvents (pure methanol, 80% ethanol, and water) for 72 h at room temperature with stirring. The extracts were then centrifuged using Hettich Universal 32 R centrifuge D-78532 (Tuttlingen, Germany) for a duration of 10 min at 4000 rpm, and filtered through filter paper blue ribbon (LabExpert, Kefo d.o.o., Ljubljana, Slovenia). Afterwards, the extracts were then rotary evaporated at 40–60 °C using a Buche rotavapor R-200 (Flawil, Switzerland) to remove methanol and ethanol, while aqueous extracts were lyophilized using a Freeze-dryer Alpha 1–4 LSCplus (Osterode am Harz, Germany). The obtained dry material was then dissolved in ultrapure water (Milli-Q water) at a stock concentration of 10 mg/mL for testing the antioxidant activity.

### 2.2. Reagents and Standards

For LC-MS/MS analysis, HPLC-grade acetonitrile was purchased from Merck KgA (Darmstadt, Germany). Water was obtained from a Milli-Q water purification system (Millipore, Billerica, MA, USA). Formic acid was purchased from Prolabo (VWR, International, France). The pure phenolic compounds, *p*-hydroxybenzoic acid, protocatechuic acid, gentisic acid, vanillic acid, gallic acid, syringic acid, *p*-coumaric acid, *o*-coumaric acid, caffeic acid, ferulic acid, chlorogenic acid, quinic acid, sinapic acid, rosmarinic acid, cinnamic acid, epicatechin, catechin, resveratrol, astringin, EGCG (Epigallocatechin gallate), hesperetin, quercetin, myricetin, apigenin, naringenin, and rutin, were obtained from Sigma-Aldrich (St. Louis, MO, USA).

### 2.3. Chemical Characterization of Phenolic Compounds

The above-mentioned *Veronica* extracts were redissolved with 20% aqueous acetonitrile to obtain a final concentration of 2 mg/mL and filtered through a 0.22 µm disposable CA syringe filter for liquid chromatography-mass spectrometry (LC-MS/MS) analysis. All samples and standards were run in triplicate. The extracts were analyzed using a mass spectrometer (SCIEX—Triple TOF 6600+) coupled to a liquid chromatography system (SCIEX—Ex-ionLC) with a binary pump. A Phenomenex Kinetex Core-Shell 2.6 µm C18 100 Å, 100 × 2.1 mm column thermostated at 40 °C was used. The solvents used were: (A) 0.1% formic acid in water and (B) acetonitrile. The elution gradient established was 5% B, 5 min 10% B, 13 min 40% B, 15 min 70% B, holding at 70% B until 16 min, 17 min 5% B, and re-equilibration at 5% B until 20 min. The flow rate was 0.3 mL/min and the injection volume was 5 µL. MS detection was performed using a Sciex QTOF 6600+ equipped with a Duo spray ion source operated in ESI mode and a quadrupole time-of-flight mass analyzer controlled by Analyst TF 1.8.1. software. The ion source parameters were as follows: GS1 = 40, GS2 = 15, curtain gas = 25, ISVF-4500, Temp = 400. The quadrupole was set to unit resolution. The MS detection method was set to perform an MS scan (TOF MS) from 100 to 700 *m*/*z* and a series of MRMHR experiments with precursors of the studied compounds chosen from the literature. The declustering potential was set to −80 for all experiments. The CE for the TOF MS experiment was −10 V, while the CE for the MRMHR experiments was optimized for each precursor and ranged between −15 and −40. The CES was set to 10. The accumulation time was 50 ms for the TOF MS scan and 45 ms for the MRMHR experiments with a resulting cycle time of 1225 ms. The compounds of interest present in the sample extracts were characterized using mass spectra and retention times determined using commercial standards. For the quantitative analysis of phenolic compounds, a calibration curve was obtained by injecting known concentrations (0.3 ng/mL–2 µg/mL) of the pure compounds. Quantification was performed using Sciex OS 1.6.1.29803 software and was based on the MS/MS peak area of the extracted ion chromatograms of selected characteristic fragments for each compound except chlorogenic acid, for which the TOF MS chromatogram of the precursor was used for quantification (Figures S1–S3). The reference standards with their precursor ions and fragments used for quantification and their retention times are given in Table 2. The concentrations of the compounds were calculated using calibration curves and the results are expressed as µg/g of dry weight (DW).

### 2.4. Antioxidant Activity of Veronica Extracts

#### 2.4.1. Measurement of the ORAC Values

An assay was performed in a Tecan Infinite 200 PRO spectrophotometer (Tecan Trading AG, Switzerland), using 96-well black polystyrene microtiter plates (Porvair Sciences, Leatherhead, UK). Each reaction contained 180 µL of fluorescein (1 µM), 70 µL 2,2′-Azobis(2-methyl-propionamidine) dihydrochloride (AAPH, Acros Organics) (300 mM), and 30 µL of blank (phosphate buffer) plant extract or reference standard Trolox (6.25–50 µM) (Sigma–Aldrich, St. Louis, MO, USA). All experimental solutions of plant extracts were prepared in a phosphate buffer (0.075 mM and pH 7.0) in a concentration of 10 mg/mL and then diluted to testing concentration, 34 µg/mL and 17 µg/mL. The ORAC values of phenolic extracts are expressed as µmol of Trolox equivalents (TEs) per g of the dry phenolic extract. The results were obtained from three independent experiments.

#### 2.4.2. Measurement of the DPPH Radical Scavenging Activity

The DPPH method for testing the antioxidant capacity of the extracts used in this study has already been described by Mensor et al. and Payet et al. [24,25], and was adapted to the plant extracts tested. This method was also performed with a Tecan Infinite 200 PRO spectrophotometer (Tecan Trading AG, Switzerland) using 96-well transparent polystyrene microtiter plates (Porvair Sciences, Leatherhead, UK). Plant extracts were used as described in the ORAC method (absolute extracts from three *Veronica* species) for the assay, but diluted to 500 µg/mL for this reaction. An amount of 100 µL of methanol (Kemika, Zagreb, Croatia) and 200 µL of the sample was pipetted into each well. Serial dilutions of the samples were prepared by pipetting 100 µL from the first row into the wells in the second row using a multichannel pipette and so on until the last row, where 100 µL of the solution was ejected after mixing. A blank sample was always added to the first column of the 96-well plates (phosphate buffer). Trolox was added to the second column and samples were added to the other columns. The reaction starts by addition of 100 µL of a DPPH (200 µM) methanolic solution to each well. The initial absorbance at 517 nm was measured immediately (it should be around 1.1). After 30 min of incubation, the absorbance was measured again and the percentage of DPPH inhibition was calculated according to the following formula by Yen and Duh [26]:

% inhibition = ((AC(0) − AA(t))/AC(0)) × 100,

where AC(0) is the absorbance of the control at t = 0 min and AA(t) is the absorbance of the antioxidant at t = 30 min. All measurements were performed in triplicate. 

Because of the data from other relevant literature, we expressed the results as IC50 values in µg of phenolic compounds/mL of phenolic solution and in Trolox equivalents.

#### 2.4.3. Measurement of the ORAC and DPPH Capacity for Standards 

The antioxidant capacity was also evaluated for four standards that were identified in all selected *Veronica* species in high quantities: apigenin, *p*-hydroxybenzoic acid, caffeic acid, and vanillic acid. The method was the same as described in the section before. The concentration of standards used for the reactions were as follows: for ORAC, 0.1 µg/mL and 0.001 µg/mL for apigenin and 0.005 µg/mL and 0.0025 µg/mL for three other standards; for DPPH, 20 µg/mL for apigenin and 4 µg/mL for other three standards.

### 2.5. Statistical Analyses 

Statistical analysis was performed in GraphPad Prism Version 9 (GraphPad Software, Inc., Boston, MA, USA). For the extraction yield of three different *Veronica* species, a two-way ANOVA test was used for statistical data processing, after which Tukey’s multiple comparison test was used to examine the difference between the same solvent for different species (*V. anagallis-aquatica*, *V. persica*, and *V. polita*) (different letter a–c), and to examine the difference between different solvents for same species (different letter d–e), *p* < 0.05. The results are presented as mean ± standard deviation (SD) (n = 2). For the identification and quantification of phenolic compounds, a one-way ANOVA test was used for statistical data processing, after which Tukey’s multiple comparison test was used to examine the difference between each individual component present in the same species extracted with three different solvents (different letter a–c), and to examine the difference between each individual component present in all investigated *Veronica* species (*V. anagallis-aquatica*, *V. persica*, and *V. polita*) extracted with same solvent (different letter d–f), *p* < 0.05. The results are presented as mean ± standard deviation (SD) (n ≥ 3). Statistical significance for phenolic compounds and antioxidant activity was assessed by multiple *t*-test. 

## 3. Results

### 3.1. LC-MS/MS Analysis of Phenolic Compounds Present in Veronica Species

The novelty of this study is the investigation of the differences in the chemical profiles of three *Veronica* species using different solvents and their influence on antioxidant activity. In addition, the pure constituents were tested for their antioxidant activity, which are the main constituents of *Veronica* species. These results are also correlated, in the discussion, with the antioxidant activity of all of the phenolic extracts. Extracts obtained by maceration in three different solvents were examined: a methanol extract, 80% ethanol extract, and water extract. Extractions were performed in duplicate (n = 2). The results were expressed in mg/g of dried plant material (Table 3) in relation to the solvents used for each *Veronica* species (*V. anagallis-aquatica*, *V. persica*, and *V. polita*). The highest extraction yield for *V. anagallis-aquatica* and *V. persica* was obtained using 80% ethanol and methanol as solvents, while the opposite was true for *V. polita*. Water as solvent gave the highest yield in contrast to 80% ethanol and methanol (Table 3). In this study, the presence and content of 26 phenolic compounds in different *Veronica* extracts were investigated using the LC/MS-MS technique (Table 4 and Figures S1–S3). The compounds *p*-hydroxybenzoic acid, protocatechuic acid, gentisic acid, vanillic acid, and caffeic acid were the most abundant phenolic acids in the *Veronica* species studied. *p*-Hydroxybenzoic acid was most abundant in *V. anagallis-aquatica* at 4.11 ± 0.02 µg/g, 5.04 ± 0.36 µg/g, and 5.70 ± 0.11 µg/g using pure methanol, 80% ethanol, and water as solvents, respectively. Of all phenolic acids, the caffeic acid content in *V. anagallis-aquatica* was highest when pure methanol and 80% ethanol were used as solvents (7.15 ± 0.20 µg/g and 7.45 ± 0.06 µg/g). The amount of caffeic acid when water was used as solvent was the lowest (1.94 ± 0.07 µg/g). Apart from *p*-hydroxybenzoic acid, the most abundant phenolic acids in *V. persica* after extraction with pure methanol, 80% ethanol, and water were protocatechuic acid (1.01 ± 0.02 µg/g, 4.79 ± 0.37 µg/g, and 3.82 ± 0.14 µg/g, respectively) and gentisic acid (1.01 ± 0.01 µg/g, 4.89 ± 0.24 µg/g, and 3.90 ± 0.05 µg/g, respectively). In this case, pure methanol proved to be the least efficient solvent. In *V. polita*, the most abundant constituent in the extracts was vanillic acid (4.24 ± 0.49 µg/g, 0.36 ± 0.04 µg/g and 4.97 ± 0.1 µg/g). The most abundant flavonoid in all three *Veronica* species was apigenin. The most efficient solvent for its extraction was 80% ethanol with an extracted content of 950.4 ± 22.4 µg/g, 661.8 ± 25.51 µg/g, and 48.53 ± 1.75 µg/g for *V. anagallis-aquatica*, *V. persica*, and *V. polita*, respectively. Water as a solvent proved to be the worst for apigenin (103.73 ± 4.16 µg/g, 27.18 ± 1.28 µg/g, and n.d.) and rutin (0.31 ± 0.03 µg/g, 0.57 ± 0.01 µg/g, and n.d.) extraction in all three *Veronica* species studied in *V. anagallis-aquatica*, *V. persica*, and *V. polita*, respectively. In general, *V. anagallis*-*aquatica* contains the highest amount of phenolic components compared to the other two species tested, *V. persica* and *V. polita* (Table 4). Overall, 80% ethanol proved to be the best solvent of all three solvents tested, considering the presence and content of phenolic components. In the further course of the study, extracts from three *Veronica* species were tested for their antioxidant activity to determine the relationship between the amount of phenolic compounds and antioxidant potential.

Extractions were performed in duplicate (n = 2). A two-way ANOVA test was used for statistical data processing, after which Tukey’s multiple comparison test was used to examine the difference between the same solvent for different species (*V. anagallis-aquatica*, *V. persica*, and *V. polita*) (different letter a–c), and to examine the difference between different solvents for same species (different letter d–e).

### 3.2. Antioxidant Activity of Phenolic Compounds Present in Studied Veronica Species

The antioxidant activity was tested using two methods: ORAC (oxygen radical absorbance capacity) and DPPH (2,2-diphenyl-1-picrylhydrazyl). We tested three extracts for three selected *Veronica* species: methanolic, 80% ethanolic, and water extracts. In addition, four standards, which were the most abundant compounds in the results for the phenolic composition, were also tested for their antioxidant activity so that the results for the better antioxidant activity of particular extracts could be supported.

#### 3.2.1. ORAC Activity

Table 5 shows that highest activity was recorded for the methanolic extract of *V. polita*, with the result of 4505.60 ± 255.34 µmol TE/g of DW. The second highest activity was determined for the *V. persica* ethanolic extract with an ORAC value of 3065.59 ± 268.73 µmol TE/g of DW. The lowest activity was tested for the *V. polita* ethanolic extract with the result of 1290.96 ± 188.07 µmol TE/g of DW. 

#### 3.2.2. DPPH Activity

In the DPPH assay (Table 6), the highest activity shown was for the methanolic extract of *V. anagallis-aquatica*, with an IC50 value of 120.19 ± 3.98 µg/mL (271.45 ± 13.53 mg TE/g of DW), which had similar activity to that exerted by the 80% ethanolic extract of *V. persica*, with an IC50 value 120.66 ± 10.90 µg/mL (268.33 ± 25.69 mg TE/g of DW). The lowest activity again resulted for the *V. polita* 80% ethanolic extract, with an IC50 value of 474.01 ± 1.45 µg/mL. 

### 3.3. Antioxidant Activity of the Standards of the Most Abundant Phenolic Compounds

Results for the antioxidant activity of the chosen standards are presented in Table 7. In both methods, caffeic acid had highest antioxidant activity, with an IC50 value in the DPPH assay of 1.99 ± 0.10 µg/mL. Also, in the ORAC method a very high value was shown, 33,057.22 ± 3312.59 mmol TE/g DW of standard. Overall, looking at the results presented in the Table 7, we can say that caffeic acid, vanillic acid, and *p*-hydroxybenzoic acid have several times higher activity than apigenin, which showed the lowest activity in both methods. This could be due to the fact that all these three compounds belong to the group of phenolic acids which are known to possess high antioxidant activity.

## 4. Discussion

Extracts obtained by maceration in three different solvents were studied: a water extract, methanol extract, and 80% ethanol extract. The best extraction yields were obtained for *V. anagallis-aquatica* and *V. persica* using MetOH and 80% EtOH solvents (Table 3). In a study by Barreira et al., similar extraction yields were reported for different *Veronica* species (*V. montana*, *V. polita*, and *V. spuria*) using an 80% methanol extract [2]. They also reported presence of caffeic acid, apigenin, and protocatechuic acid [2]. Stojković et al. also reported protocatechuic acid as a major compound in *V. montana* [27]. 

This research involved the identification and quantification of phenolic compounds in different *Veronica* extracts using the LC/MS-MS technique. The results showed that the main components in all extracts of the tested *Veronica* species (*V. anagallis aquatica*, *V. persica*, and *V. polita*) were as follows: *p*-hydroxybenzoic acid, protocatechuic acid, gentisic acid, vanillic acid, caffeic acid, and apigenin. These results are consistent with Beara et al., who also reported a high amount of chlorogenic acid in 70% aqueous acetone extracts of three *Veronica* species (*V. teucrium*, *V. jacquini*, and *V. urticifolia*) [28]. A potential explanation for the differences in the chemical composition lies in the different extraction solvent used and different investigated *Veronica* species than those reported by Beara et al. [28]. 

Comparing the LC-MS/MS results for phenolic compound composition and antioxidant activity that the extracts had, several connections can be drawn. First, apigenin is present in all plant extracts in the highest concentration when comparing to other identified compounds. Also, the 80% ethanolic solution extracted the highest number and quantity of phenolic acids that, referring to the literature data, have the highest antioxidant potential of all phenolics present in plants. The highest activity among the tested standards was shown for caffeic and vanillic acid, and these results can be correlated with the results for the difference in antioxidant activity of the tested samples. The methanolic and ethanolic extracts of *V. anagallis aquatica* that have the highest quantity of caffeic acid showed the highest and second highest antioxidant activity in the DPPH method, so this high amount of caffeic acid could be the reason for this activity. These extracts also have very high amounts of apigenin, so although this standard showed the lowest antioxidant activity among standards, due to its very high amounts in these extracts, it probably also had an impact on the antioxidant activity. Another interesting finding is for *V. polita*, where the water extract had higher antioxidant activity than the ethanolic extract. In Table 4, it can be seen that this water extract has high amounts of vanillic acid compared to other compounds, so this could be the reason. For the *V. persica* species, the ethanolic extract showed the second highest activity in the ORAC method, which could be due to higher amounts of caffeic acid and apigenin (Table 5). 

Other findings for the antioxidant activity of *Veronica* species reported variable results due to using different solvents and extraction methods. In a study by Ertas et al., a methanolic extract of *Veronica thymoides* ssp. *pseudocinerea* also showed higher DPPH activity than a water extract, but this species had higher activity than all three species tested in our study [13]. Sharifi-Rad et al. reported DPPH antioxidant activity for a methanolic extract of 30 µg/mL which is four to ten times higher than the results in this study. This could be due to different extraction and measurement methods and the fact that the plant grew in different conditions and probably had a different phenolic composition [29]. Živković et al. [17] reported higher DPPH antioxidant activity for *V. teucrium* and *V. jacquinii*, 28.49 ± 0.6 µg/mL and 37.63 ± 0.6 µg/mL, respectively. Harput et al. reported DPPH antioxidant activity for methanolic extracts of *V. officinalis*, *V. peduncularis*, *V. orientalis*, and *V. baranetzkii*. The IC50 values were 37.68 µg/mL, 54.19 µg/mL, 85.1 µg/mL, and 99.03 µg/mL, respectively [30]. In another study by Harput et al., they reported the DPPH antioxidant activity for a methanolic extract of *V. persica* to be 34 µg/mL [31]. In a study by Dunkić et al. [3], methanolic and ethyl-acetate extracts of *V. spicata* showed substantial antioxidant activity, especially the methanol extracts of flowers and leaves, with IC50 values of 8.21 µg/mL and 8.69 µg/mL, respectively [29]. In a study by Nikolova M., DPPH activity higher than 200 µg/mL was tested for *V. urticifolia* and *V. serpyllifolia*, but for other *Veronica* species like *V. officinalis*, *V. vindobonensis*, and *V. chamaedrys*, higher DPPH activity was reported that is comparable to other medicinal plants such as *Clinopodium vulgarae*, *Clematis vitalba*, and *Stachys recta* [32]. All these differences in reported antioxidant activities are probably due to the fact that the majority of the reported tested extracts were prepared by dissolving dry methanolic extract in DMSO solution, and in our study we dissolved dry phenolic extracts in pure water so that the same extracts could be used in another future tests such as for cytotoxic and antibacterial activities. This way, some amount of phenolic compounds was probably lost (not dissolved) and therefore the DPPH activity of some of our extracts is somewhat lower than previously reported. ORAC values are lower than the values for some of the most commonly used medicinal herbs such as *Salvia officinalis*, *Satureja* sp., and *Thymus* sp., but they are similar to the species such as *Rosmarinus officinalis*, as reported in Ninfali et al. [33]. Zheng et al. reported ORAC values for 27 culinary herbs and 12 medicinal herbs. The ORAC value for *V. polita* methanolic extract was similar to the one reported for mexican oregano (*Poliomintha longiflora*), 5121 µg/g phenolic compounds [34]. Wojcikowski et al. reported ORAC values for 55 medicinal herbs used for treatment of the urinary system and according to these values, the ORAC activity of all three species tested in our study is higher than the herbs reported in the mentioned work [35]. All these results show that speedwells and their phenolic extracts should be further researched for their in vivo/in vitro antioxidant activities for potential usage as natural products in different aspects, such as in the pharmaceutical industry, cosmetic products, and food preservations.

## 5. Conclusions

In this study, the phenolic profiles and antioxidant activity of *Veronica anagallis-aquatica*, *V. persica*, and *V. polita* were investigated. The main phenolic compounds present in all three *Veronica* species studied were as follows: *p*-hydroxybenzoic acid, apigenin (with the exception of the *V. polita* water extract), vanillic acid, caffeic acid, protocatechuic, and gentisic acid. The pure compound caffeic acid showed the highest antioxidant activity in both methods tested. As for the plant extracts, methanolic or 80% ethanolic extracts showed higher activity than water extracts in both methods, which was expected as organic solutions extract more phenolic compounds. As a result of this study, it can be concluded that the use of different solvents affects the phenolic yield and chemical composition of extracts from *Veronica* species and thus their antioxidant activity. The limitations of the study include studying one extraction technique using air drying for the plant material. Further studies should include investigating different drying and extraction techniques, to finally conclude which solvents and techniques will extract the most wanted compounds. 

## Figures and Tables

**Table 1:** Details on collection data and origin of the three *Veronica* species studied.

Taxa	Locality	Latitude	Longitude	Altitude a.s.l. (m)	Voucher No.
*V. anagallis-aquatica* L.	Split	43°31′43.5″ N	16°28′45.2″ E	22	CROVeS-06-2021
*V. persica* Poir.	Hvar	43°09′42.8″ N	16°40′37.6″ E	18	CROVeS-22-2021
*V. polita* Fr.	Hvar	43°10′42.3″ N	16°36′43.6″ E	38	CROVeS-23-2021

**Table 2:** Precursor and quantitation fragments *m*/*z* and retention time of phenolic compounds.

Compound	Precursor *m*/*z*	Fragment *m*/*z*	t_r_ (min)
*p*-hydroxybenzoic acid	137.02	93.03	3.48
Protocatechuic acid	153.02	109.03	2.21
Gentisic acid	153.02	109.03	3.21
Vanillic acid	167.03	152.01	4.66
Gallic acid	169.01	125.02	1.33
Syringic acid	197.04	123.01	5.59
*p*-coumaric acid	163.04	119.05	7.18
*o*-coumaric acid	163.04	119.05	9.16
Caffeic acid	179.03	135.04	4.9
Ferulic acid	193.05	134.03	8.19
Chlorogenic acid	353.08	179.03	4.64
Quinic acid	191.05	85.03	4.64
Sinapic acid	223.06	193.01	8.49
Rosmarinic acid	359.08	161.02	9.88
Cinnamic acid	147.05	103.06	11.17
Epicatechin	289.07	109.03	6.25
Catechin	289.07	109.03	4.17
Resveratrol	227.07	143.05	10.42
Astringin	405.12	243.06	7.50
EGCG (Epigallocatechin gallate)	457.08	169.01	6.95
Hesperetin	301.07	164.01	12.61
Quercetin	301.03	151.00	11.36
Myricetin	317.03	151.00	10.03
Apigenin	269.04	117.03	12.46
Naringenin	271.06	151.00	12.14
Rutin	609.15	300.03	8.86

**Table 3:** Extraction yield of three different *Veronica* species.

Extracts	*V. anagallis-aquatica*	*V. persica*	*V. polita*
Extraction yield (%)			
MetOH extract	26.00 ± 2.83 ^a,d^	18.25 ± 3.18 ^b,d^	11.50 ± 2.12 ^c,d^
80% EtOH extract	32.00 ± 1.41 ^a,d^	18.00 ± 0.00 ^b,d^	14..00 ± 2.83 ^b,d^
Water extract	8.00 ± 1.41 ^a,e^	10.25 ± 1.77 ^a,e^	15.50 ± 2.12 ^b,a,d^

**Table 4:** Identification and quantification of the phenolic compounds in different extracts of three *Veronica* species (*V. anagallis-aquatica*, *V. persica*, and *V. polita*).

	*V. anagallis-aquatica*			*V. persica*			*V. polita*		
Compound	MetOH Extract	80% EtOH Extract	Water Extract	MetOH Extract	80% EtOH Extract	Water Extract	MetOH Extract	80% EtOH Extract	Water Extract
(µg/g of DW)									
*p*-hydroxybenzoic acid	4.11 ± 0.02 ^a,d^	5.04 ± 0.36 ^b,d^	5.70 ± 0.11 ^c,d^	0.61 ± 0.01 ^a,e^	2.91 ± 0.16 ^b,e^	1.41 ± 0.07 ^c,e^	0.66 ± 0.02 ^a,f^	0.54 ± 0.02 ^b,f^	0.28 ± 0.02 ^c,f^
Protocatechuic acid	3.44 ± 0.07 ^a,d^	3.81 ± 0.13 ^b,d^	4.84 ± 0.05 ^c,d^	1.01 ± 0.02 ^a,e^	4.79 ± 0.37 ^b,e^	3.82 ± 0.14 ^c,e^	0.08 ± 0.01 ^a,f^	0.24 ± 0.004 ^b,f^	0.24 ± 0.01 ^b,f^
Gentisic acid	3.41 ± 0.01 ^a,d^	3.84 ± 0.20 ^b,d^	4.98 ± 0.07 ^c,d^	1.01 ± 0.01 ^a,e^	4.89 ± 0.24 ^b,e^	3.90 ± 0.05 ^c,e^	0.11 ± 0.00 ^a,f^	0.27 ± 0.01 ^b,f^	0.28 ± 0.01 ^b,f^
Vanillic acid	2.73 ± 0.19 ^a,d^	2.99 ± 0.18 ^a,d^	3.45 ± 0.35 ^b,d^	0.79 ± 0.03 ^a,e^	1.98 ± 0.15 ^b,e^	0.76 ± 0.04 ^a,e^	4.24 ± 0.49 ^a,f^	0.36 ± 0.04 ^b,f^	4.97 ± 0.12 ^c,f^
Gallic acid	n.d. ^a,d^	0.38 ± 0.01 ^a,d^	n.d. ^a^	n.d. ^a,d^	1.11 ± 0.08 ^b,e^	n.d. ^a^	n.d. ^a,d^	0.18 ± 0.002 ^b,f^	n.d. ^a^
Syringic acid	n.d. ^a,d^	n.d. ^a^	0.11 ± 0.01 ^a,d^	0.26 ± 0.00 ^a,e^	n.d. ^b^	0.17 ± 0.00 ^c,e^	0.18 ± 0.00 ^a,f^	n.d. ^b^	0.37 ± 0.00 ^c,f^
*p*-coumaric acid	1.03 ± 0.04 ^a,d^	1.25 ± 0.01 ^a,d^	0.53 ± 0.02 ^b,d^	0.22 ± 0.00 ^a,e^	0.64 ± 0.02 ^b,e^	0.33 ± 0.01 ^c,e^	0.81 ± 0.02 ^a,f^	1.69 ± 0.08 ^b,f^	0.08 ± 0.00 ^c,f^
*o*-coumaric acid	n.d.	n.d. ^d^	n.d.	n.d. ^a^	n.d. ^d^	n.d.	n.d. ^a^	0.09 ± 0.001 ^b,e^	n.d. ^a^
Caffeic acid	7.15 ± 0.20 ^a,d^	7.45 ± 0.06 ^a,d^	1.94 ± 0.07 ^a,d^	0.32 ± 0.01 ^a,e^	3.88 ± 0.04 ^b,e^	1.42 ± 0.05 ^c,e^	0.08 ± 0.00 ^a,f^	1.24 ± 0.04 ^b,f^	0.05 ± 0.00 ^a,f^
Ferulic acid	0.50 ± 0.02 ^a,d^	0.54 ± 0.02 ^a,d^	0.18 ± 0.001 ^b,d^	0.35 ± 0.03 ^a,e^	0.30 ± 0.01 ^a,e^	0.78 ± 0.02 ^b,e^	0.56 ± 0.02 ^a,d^	0.14. ± 0.00 ^b,f^	0.13 ± 0.00 ^b,f^
Chlorogenic acid	0.65 ± 0.03 ^a,d^	2.96 ± 0.01 ^b,d^	0.16 ± 0.001 ^c,d^	1.60 ± 0.01 ^a,e^	0.26 ± 0.01 ^b,e^	1.06 ± 0.02 ^c,e^	0.17 ± 0.00 ^a,f^	0.40 ± 0.01 ^b,f^	0.20 ± 0.00 ^c,f^
Quinic acid	n.d. ^d^	n.d.	n.d.	1.54 ± 0.13 ^a,e^	n.d. ^b^	n.d. ^b^	n.d. ^d^	n.d.	n.d.
Sinapic acid	n.d.	n.d.	n.d. ^d^	n.d.	n.d.	n.d. ^d^	n.d. ^a^	n.d. ^a^	0.56 ± 0.06 ^b,e^
Rosmarinic acid	n.d.	n.d.	n.d.	n.d.	n.d.	n.d.	n.d.	n.d.	n.d.
Cinnamic acid	n.d.	n.d.	n.d. ^d^	n.d.	n.d.	n.d. ^d^	n.d. ^a^	n.d. ^a^	0.59 ± 0.02 ^b,e^
Epicatechin	n.d.	n.d.	n.d.	n.d.	n.d.	n.d.	n.d.	n.d.	n.d.
Catechin	n.d.	n.d.	n.d.	n.d.	n.d.	n.d.	n.d.	n.d.	n.d.
Resveratrol	n.d.	n.d.	n.d.	n.d.	n.d.	n.d.	n.d.	n.d.	n.d.
Astringin	n.d.	n.d.	n.d.	n.d.	n.d.	n.d.	n.d.	n.d.	n.d.
EGCG (Epigallocatechin gallate)	n.d.	n.d.	n.d.	n.d.	n.d.	n.d.	n.d.	n.d.	n.d.
Hesperetin	n.d.	n.d.	n.d.	n.d.	n.d.	n.d.	n.d.	n.d.	n.d.
Quercetin	2.15 ± 0.07 ^a,d^	3.24 ± 0.22 ^b,d^	n.d. ^a,d^	n.d. ^a,e^	2.21 ± 0.13 ^b,e^	4.22 ± 0.55 ^c,e^	n.d. ^a,e^	1.16 ± 0.02 ^b,f^	n.d. ^a,d^
Myricetin	n.d.	n.d.	n.d.	n.d.	n.d.	n.d.	n.d.	n.d.	n.d.
Apigenin	339.65 ± 25.94 ^a,d^	950.4 ± 22.4 ^b,d^	103.73 ± 4.16 ^c,d^	76.56 ± 3.23 ^a,e^	661.8 ± 25.51 ^b,e^	27.18 ± 1.28 ^c,e^	5.31 ± 0.13 ^a,f^	48.53 ± 1.75 ^b,f^	n.d. ^c,f^
Naringenin	0.33 ± 0.00 ^a,d^	0.64 ± 0.03 ^b,d^	0.19 ± 0.00 ^c,d^	0.23 ± 0.001 ^a,e^	0.56 ± 0.02 ^b,e^	0.11 ± 0.001 ^c,e^	0.17 ± 0.001 ^a,f^	0.22 ± 0.01 ^b,f^	0.21 ± 0.01 ^b,f^
Rutin	5.47 ± 0.63 ^a,d^	3.52 ± 0.17 ^b,d^	0.31 ± 0.03 ^c,d^	2.33 ± 0.23 ^e^	1.04 ± 0.07 ^a,e^	0.57 ± 0.01 ^b,e^	n.d. ^a,c,f^	0.59 ± 0.03 ^b,f^	n.d. ^a,f^

Legend: n.d.—not detected; dw—dry weight. Data are presented as mean ± SD, n = 3. A one-way ANOVA test was used for statistical data processing, after which Tukey’s multiple comparison test was used to examine the difference between each individual component present in the same species extracted with three different solvents (different letter a–c), and to examine the difference between each individual component present in all investigated *Veronica* species (*V. anagallis-aquatica*, *V. persica*, and *V. polita*) extracted with same solvent (different letter d–f).

**Table 5:** Antioxidant activity of three extracts (methanolic, 80% ethanolic, and water) of the chosen *Veronica* species in ORAC method.

Extract/Species	*V. anagallis-aquatica*	*V. persica*	*V. polita*
Methanolic	2678.40 ± 86.88 ^Ab^	2169.58 ± 131.00 ^Bc^	4505.60 ± 255.34 ^Aa^
Ethanolic 80%	2652.49 ± 366.17 ^Ab^	3065.59 ± 268.73 ^Aa^	1290.96 ± 188.07 ^Cc^
Water	1339.84 ± 126.23 ^Bb^	1561.02 ± 28.01 ^Cb^	1810.31 ± 37.53 ^Ba^

ORAC, oxygen radical absorbance capacity, results for phenolic extracts expressed as µmol of Trolox equivalents (TEs) per g of DW of extracted phenolic compounds; a two-way ANOVA test was used for statistical data processing (denoted with letters a–c in the rows) comparing antioxidant activity between different species, after which Tukey’s multiple comparison test was used to examine the difference in antioxidant activity in the same species extracted with three different solvents (denoted with capital letter A–C in the columns); activities with the same letters in the rows (a–c) meaning that there is no statistical significance, and activities with the same letters in the columns (A–C) meaning that there is no statistical significance.

**Table 6:** Antioxidant activity of three extracts (methanolic, 80% ethanolic, and water) of the chosen *Veronica* species in DPPH method.

	Extract/Species	*V. anagallis-aquatica*	*V. persica*	*V. polita*
IC50	Methanolic	120.19 ± 3.98 ^Aa^	180.57 ± 25.34 ^Ba^	145.76 ± 24.02 ^Aa^
	Ethanolic 80%	126.72 ± 17.27 ^Aa^	120.65 ± 10.90 ^Aa^	474.01 ± 1.45 ^Cb^
	Water	198.99 ± 14.62 ^Ba^	228.95 ± 16.32 ^Ca^	257.17 ± 19.26 ^Ba^
Trolox Equivalents	Methanolic	271.45 ± 13.53 ^Aa^	170.67 ± ±24.01 ^Ba^	178.38 ± 2.83 ^Aa^
	Ethanolic 80%	254.67 ± 39.70 ^Aa^	268.33 ± 25.69 ^Aa^	85.33 ± 12.86 ^Bb^
	Water	160.50 ± 6.36 ^Ba^	146.33 ± 12.70 ^Ba^	140.22 ± 16.82 ^Aa^

DPPH results for phenolic extracts expressed as IC50 in µg/m and in Trolox Equivalents (TEs), mg TE/g DW of the extract; SD = standard deviation of triplicate analysis; a two-way ANOVA test was used for statistical data processing (denoted with letters a,b in the rows) comparing antioxidant activity between different species, after which Tukey’s multiple comparison test was used to examine the difference in antioxidant activity in the same species extracted with three different solvents (denoted with capital letter A–C in the columns); activities with the same letters in the rows (a,b) meaning that there is no statistical significance, and activities with the same letters in the columns (A–C) meaning that there is no statistical significance.

**Table 7:** Antioxidant activity of four chosen standards in ORAC and DPPH method.

Standard/Method	ORAC	DPPH
Apigenin	438.90 ± 46.52 ^c^	17.91 ± 3.87 ^a^
*p*-hydroxybenzoic acid	32,519.03 ± 3568.44 ^a^	3.79 ± 0.44 ^a^
Caffeic acid	33,057.22 ± 3312.59 ^a^	1.99 ± 0.10 ^a^
Vanillic acid	28,072.31 ± 1815.50 ^b^	2.92 ± 0.47 ^a^

For ORAC, results are expressed as mmol of Trolox equivalents (TEs) per g of dry weight of standard; for DPPH, results are expressed as the IC50 value in µg/mL; SD = standard deviation of triplicate analysis; a two-way ANOVA test was used for statistical data processing (denoted with letters a–c in the columns) comparing antioxidant activity between different standards for each method separately.

## Data Availability

The samples and any additional research data are available from the authors on request.

## Supplementary Materials

The following supporting information can be downloaded at: https://www.mdpi.com/article/10.3390/antiox13060738/s1. Figure S1. Chromatograms of *V. anagallis-aquatica*, (a) methanolic extract, (b) ethanolic extract, (c) water extract. Figure S2. Chromatograms of *V. persica*, (a) methanolic extract, (b) ethanolic extract, (c) water extract. Figure S3. Chromatograms of *V. polita*, (a) methanolic extract, (b) ethanolic extract, (c) water extract.
