# Supplementary material for: Identification of Phenolic Compounds Present in Three Speedwell (Veronica L.) Species and Their Antioxidant Potential
Source: Antioxidants (Basel). 2024 Jun 17;13(6):738. doi: 10.3390/antiox13060738 (PMC11200581; doi:10.3390/antiox13060738)
Supplement: Supplementary file 1 [file antioxidants-13-00738-s001.zip › antioxidants-3008722-supplementary.pdf]

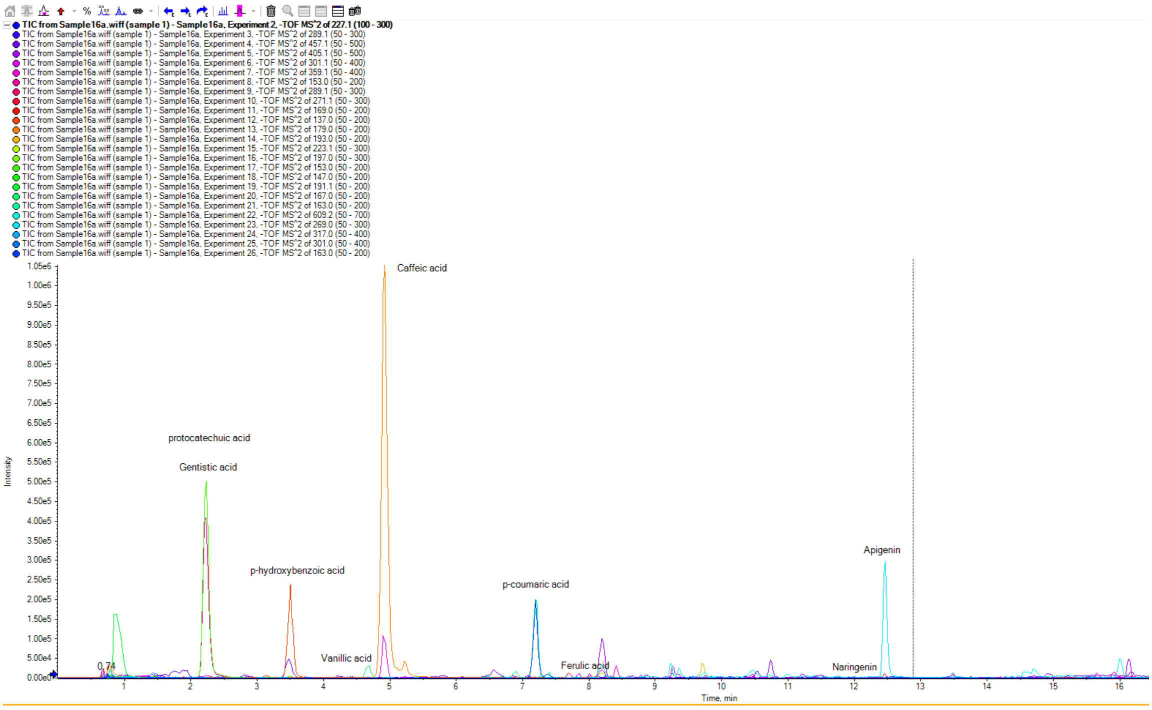
Caffeic acid
protocatechuic acid
Gentistic acid
p-hydroxybenzoic acid
Vanillic acid
p-coumaric acid
Ferulic acid
Naringenin
Apigenin
0.74
Intensity
Time, min

(a)

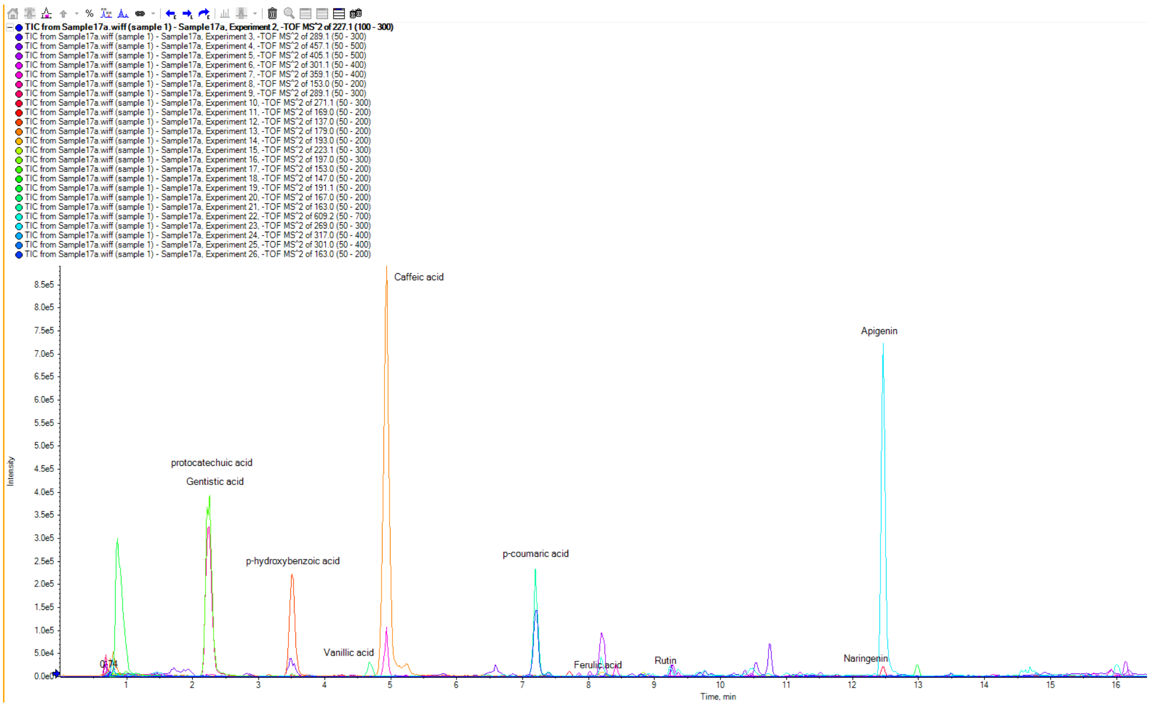
Caffeic acid
Apigenin
protocatechuic acid
Gentistic acid
p-hydroxybenzoic acid
p-coumaric acid
Vanillic acid
Ferulic acid
Rutin
Naringenin
0.74
Intensity
Time, min

(b)

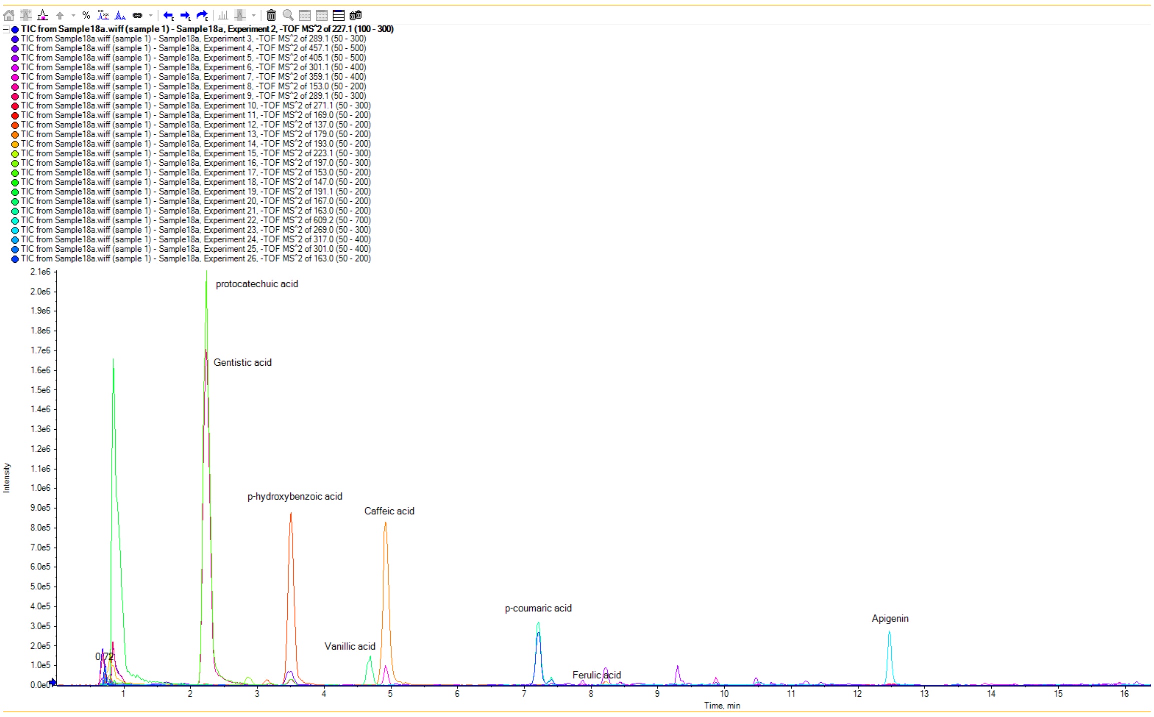

(**c**)

Figure S1. Chromatograms of *V. anagallis-aquatica*, (**a**) methanolic extract, (**b**) ethanolic extract, (**c**) water extract.

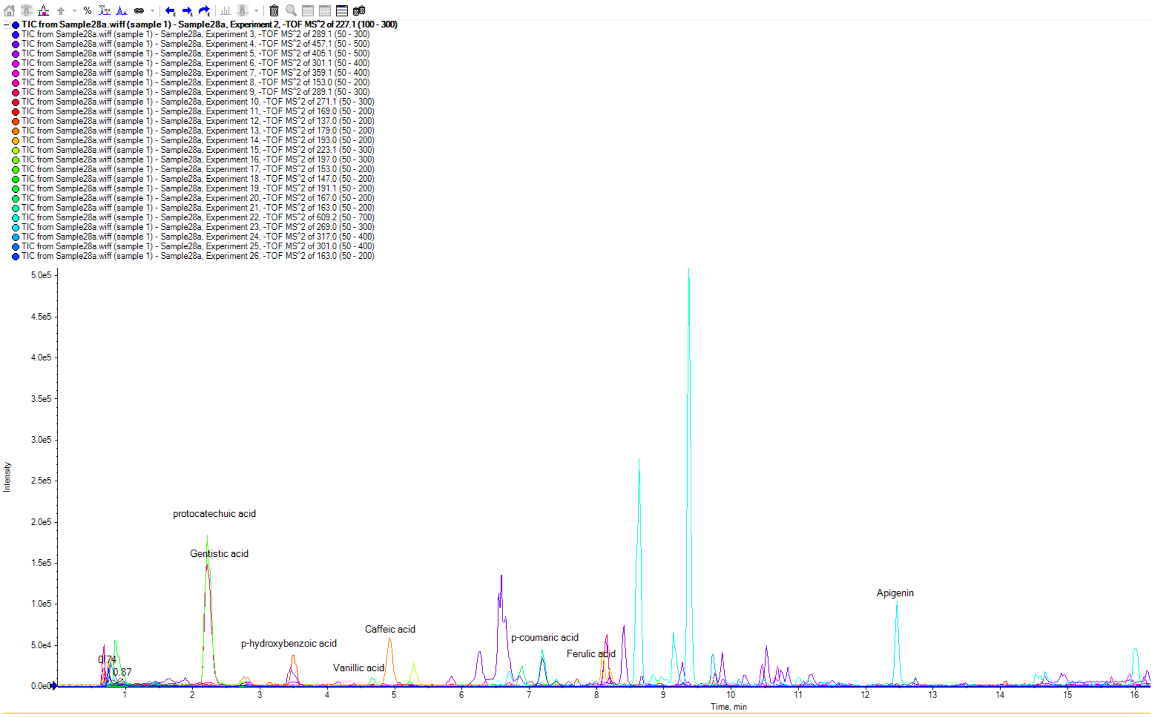
TIC from Sample28a.wiff (sample 1) - Sample28a, Experiment 2, -TOF MS^2 of 227.1 (100 - 300)
TIC from Sample28a.wiff (sample 1) - Sample28a, Experiment 3, -TOF MS^2 of 289.1 (50 - 300)
TIC from Sample28a.wiff (sample 1) - Sample28a, Experiment 4, -TOF MS^2 of 457.1 (50 - 500)
TIC from Sample28a.wiff (sample 1) - Sample28a, Experiment 5, -TOF MS^2 of 405.1 (50 - 500)
TIC from Sample28a.wiff (sample 1) - Sample28a, Experiment 6, -TOF MS^2 of 301.1 (50 - 400)
TIC from Sample28a.wiff (sample 1) - Sample28a, Experiment 7, -TOF MS^2 of 359.1 (50 - 400)
TIC from Sample28a.wiff (sample 1) - Sample28a, Experiment 8, -TOF MS^2 of 153.0 (50 - 200)
TIC from Sample28a.wiff (sample 1) - Sample28a, Experiment 9, -TOF MS^2 of 289.1 (50 - 300)
TIC from Sample28a.wiff (sample 1) - Sample28a, Experiment 10, -TOF MS^2 of 271.1 (50 - 300)
TIC from Sample28a.wiff (sample 1) - Sample28a, Experiment 11, -TOF MS^2 of 169.0 (50 - 200)
TIC from Sample28a.wiff (sample 1) - Sample28a, Experiment 12, -TOF MS^2 of 137.0 (50 - 200)
TIC from Sample28a.wiff (sample 1) - Sample28a, Experiment 13, -TOF MS^2 of 179.0 (50 - 200)
TIC from Sample28a.wiff (sample 1) - Sample28a, Experiment 14, -TOF MS^2 of 193.0 (50 - 200)
TIC from Sample28a.wiff (sample 1) - Sample28a, Experiment 15, -TOF MS^2 of 223.1 (50 - 300)
TIC from Sample28a.wiff (sample 1) - Sample28a, Experiment 16, -TOF MS^2 of 197.0 (50 - 300)
TIC from Sample28a.wiff (sample 1) - Sample28a, Experiment 17, -TOF MS^2 of 153.0 (50 - 200)
TIC from Sample28a.wiff (sample 1) - Sample28a, Experiment 18, -TOF MS^2 of 147.0 (50 - 200)
TIC from Sample28a.wiff (sample 1) - Sample28a, Experiment 19, -TOF MS^2 of 191.1 (50 - 200)
TIC from Sample28a.wiff (sample 1) - Sample28a, Experiment 20, -TOF MS^2 of 167.0 (50 - 200)
TIC from Sample28a.wiff (sample 1) - Sample28a, Experiment 21, -TOF MS^2 of 163.0 (50 - 200)
TIC from Sample28a.wiff (sample 1) - Sample28a, Experiment 22, -TOF MS^2 of 609.2 (50 - 700)
TIC from Sample28a.wiff (sample 1) - Sample28a, Experiment 23, -TOF MS^2 of 269.0 (50 - 300)
TIC from Sample28a.wiff (sample 1) - Sample28a, Experiment 24, -TOF MS^2 of 317.0 (50 - 400)
TIC from Sample28a.wiff (sample 1) - Sample28a, Experiment 25, -TOF MS^2 of 301.0 (50 - 400)
TIC from Sample28a.wiff (sample 1) - Sample28a, Experiment 26, -TOF MS^2 of 163.0 (50 - 200)
Intensity
Time, min
protocatechuic acid
Gentistic acid
p-hydroxybenzoic acid
Vanillic acid
Caffeic acid
p-coumaric acid
Ferulic acid
Apigenin
0.74
0.87

(**a**)

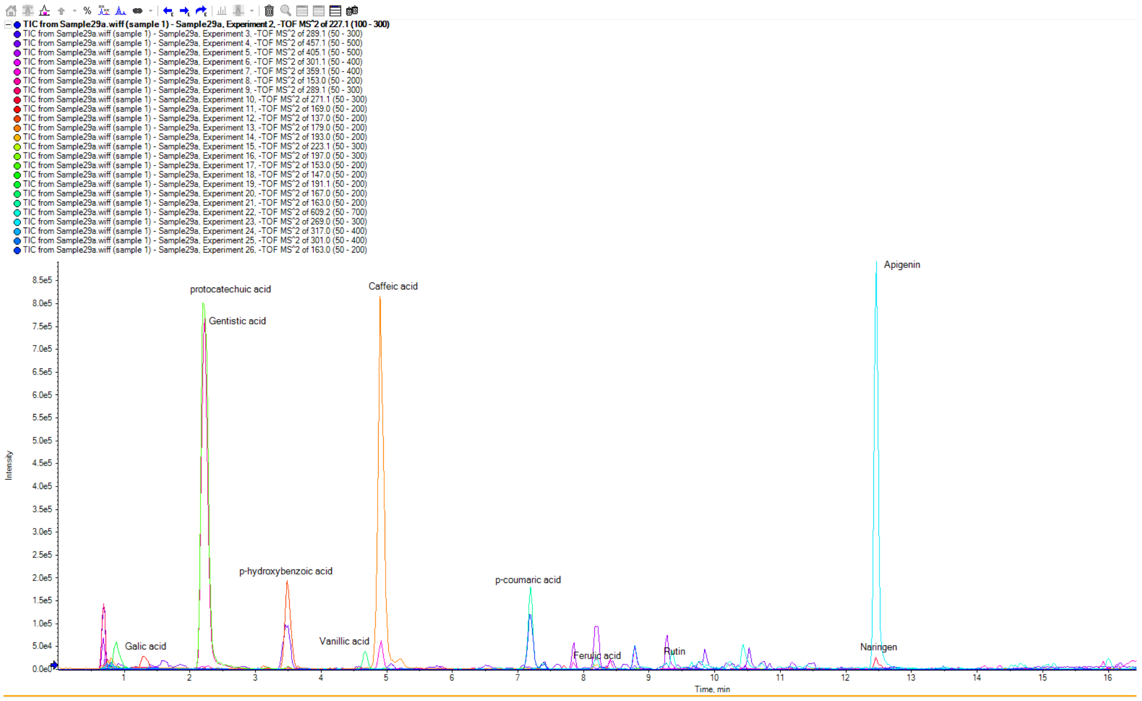
TIC from Sample29a.wiff (sample 1) - Sample29a, Experiment 2, -TOF MS^2 of 227.1 (100 - 300)
TIC from Sample29a.wiff (sample 1) - Sample29a, Experiment 3, -TOF MS^2 of 289.1 (50 - 300)
TIC from Sample29a.wiff (sample 1) - Sample29a, Experiment 4, -TOF MS^2 of 457.1 (50 - 500)
TIC from Sample29a.wiff (sample 1) - Sample29a, Experiment 5, -TOF MS^2 of 405.1 (50 - 500)
TIC from Sample29a.wiff (sample 1) - Sample29a, Experiment 6, -TOF MS^2 of 301.1 (50 - 400)
TIC from Sample29a.wiff (sample 1) - Sample29a, Experiment 7, -TOF MS^2 of 359.1 (50 - 400)
TIC from Sample29a.wiff (sample 1) - Sample29a, Experiment 8, -TOF MS^2 of 153.0 (50 - 200)
TIC from Sample29a.wiff (sample 1) - Sample29a, Experiment 9, -TOF MS^2 of 289.1 (50 - 300)
TIC from Sample29a.wiff (sample 1) - Sample29a, Experiment 10, -TOF MS^2 of 271.1 (50 - 300)
TIC from Sample29a.wiff (sample 1) - Sample29a, Experiment 11, -TOF MS^2 of 169.0 (50 - 200)
TIC from Sample29a.wiff (sample 1) - Sample29a, Experiment 12, -TOF MS^2 of 137.0 (50 - 200)
TIC from Sample29a.wiff (sample 1) - Sample29a, Experiment 13, -TOF MS^2 of 179.0 (50 - 200)
TIC from Sample29a.wiff (sample 1) - Sample29a, Experiment 14, -TOF MS^2 of 193.0 (50 - 200)
TIC from Sample29a.wiff (sample 1) - Sample29a, Experiment 15, -TOF MS^2 of 223.1 (50 - 300)
TIC from Sample29a.wiff (sample 1) - Sample29a, Experiment 16, -TOF MS^2 of 197.0 (50 - 300)
TIC from Sample29a.wiff (sample 1) - Sample29a, Experiment 17, -TOF MS^2 of 153.0 (50 - 200)
TIC from Sample29a.wiff (sample 1) - Sample29a, Experiment 18, -TOF MS^2 of 147.0 (50 - 200)
TIC from Sample29a.wiff (sample 1) - Sample29a, Experiment 19, -TOF MS^2 of 191.1 (50 - 200)
TIC from Sample29a.wiff (sample 1) - Sample29a, Experiment 20, -TOF MS^2 of 167.0 (50 - 200)
TIC from Sample29a.wiff (sample 1) - Sample29a, Experiment 21, -TOF MS^2 of 163.0 (50 - 200)
TIC from Sample29a.wiff (sample 1) - Sample29a, Experiment 22, -TOF MS^2 of 609.2 (50 - 700)
TIC from Sample29a.wiff (sample 1) - Sample29a, Experiment 23, -TOF MS^2 of 269.0 (50 - 300)
TIC from Sample29a.wiff (sample 1) - Sample29a, Experiment 24, -TOF MS^2 of 317.0 (50 - 400)
TIC from Sample29a.wiff (sample 1) - Sample29a, Experiment 25, -TOF MS^2 of 301.0 (50 - 400)
TIC from Sample29a.wiff (sample 1) - Sample29a, Experiment 26, -TOF MS^2 of 163.0 (50 - 200)
Intensity
Time, min
protocatechuic acid
Gentistic acid
Caffeic acid
Apigenin
p-hydroxybenzoic acid
p-coumaric acid
Galic acid
Vanillic acid
Ferulic acid
Rutin
Naringen

(**b**)

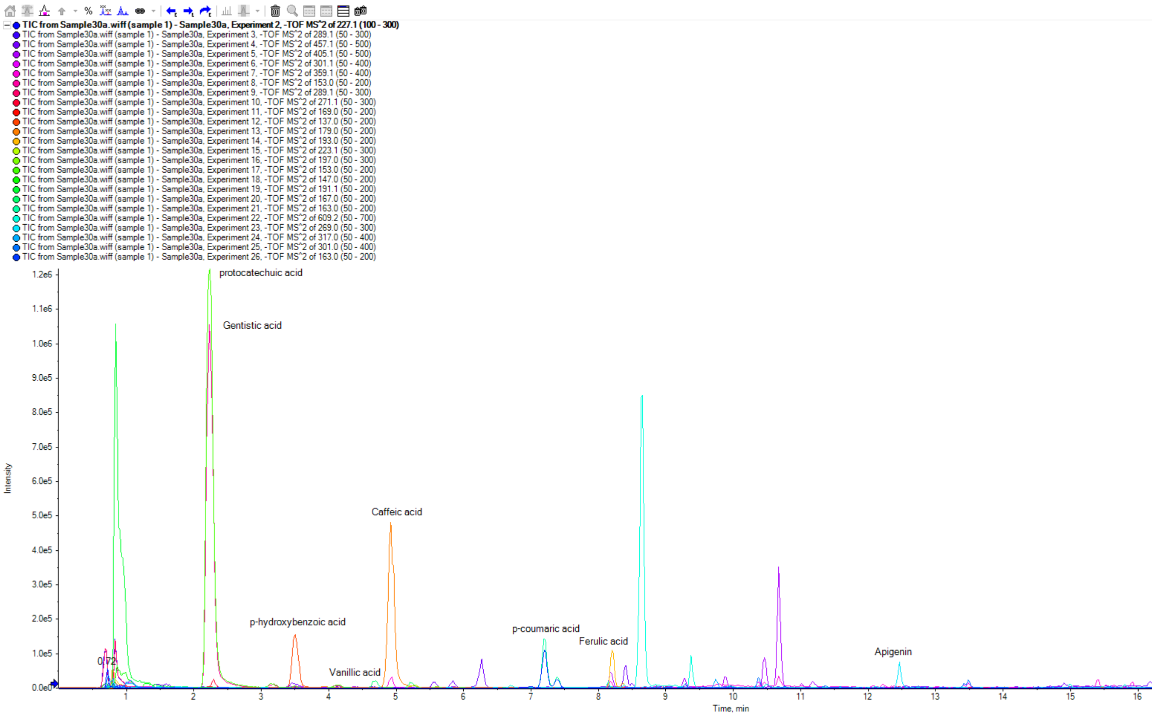

(**c**)

Figure S2. Chromatograms of *V. persica*, (**a**) methanolic extract, (**b**) ethanolic extract, (**c**) water extract.

(a)

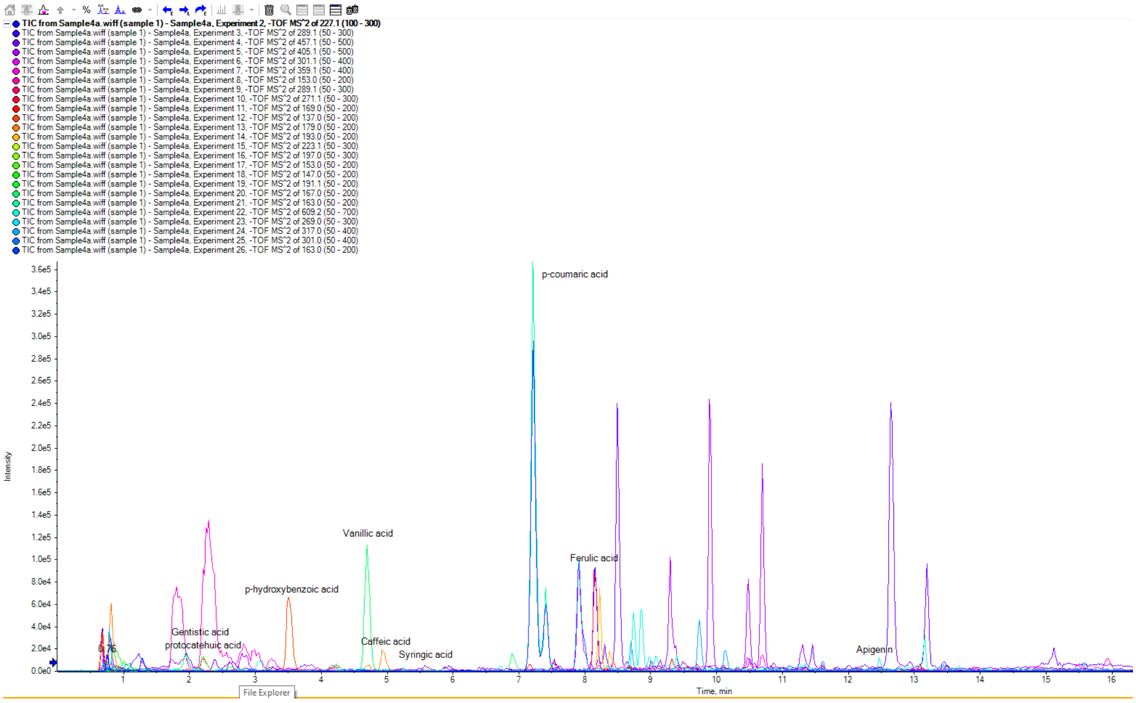

p-coumaric acid
Vanillic acid
Ferulic acid
p-hydroxybenzoic acid
Gentistic acid
protocatehuic acid
Caffeic acid
Syringic acid
Apigenin
Intensity
Time, min
File Explorer

(b)

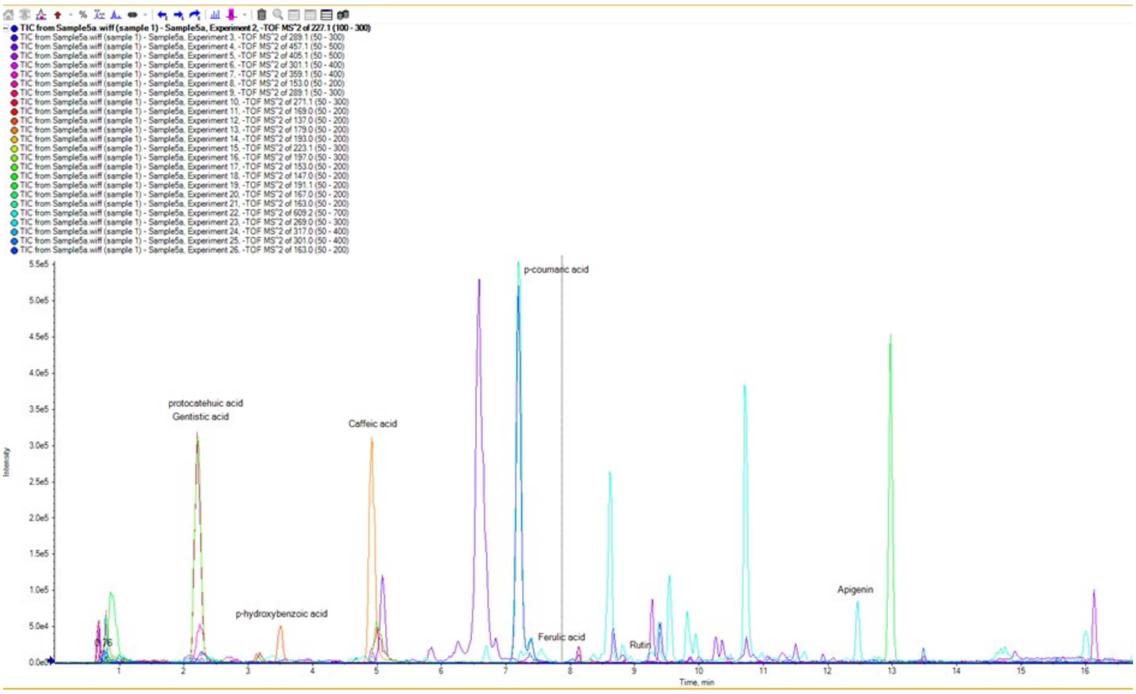

p-coumaric acid
protocatehuic acid
Gentistic acid
Caffeic acid
p-hydroxybenzoic acid
Ferulic acid
Rutin
Apigenin
Intensity
Time, min

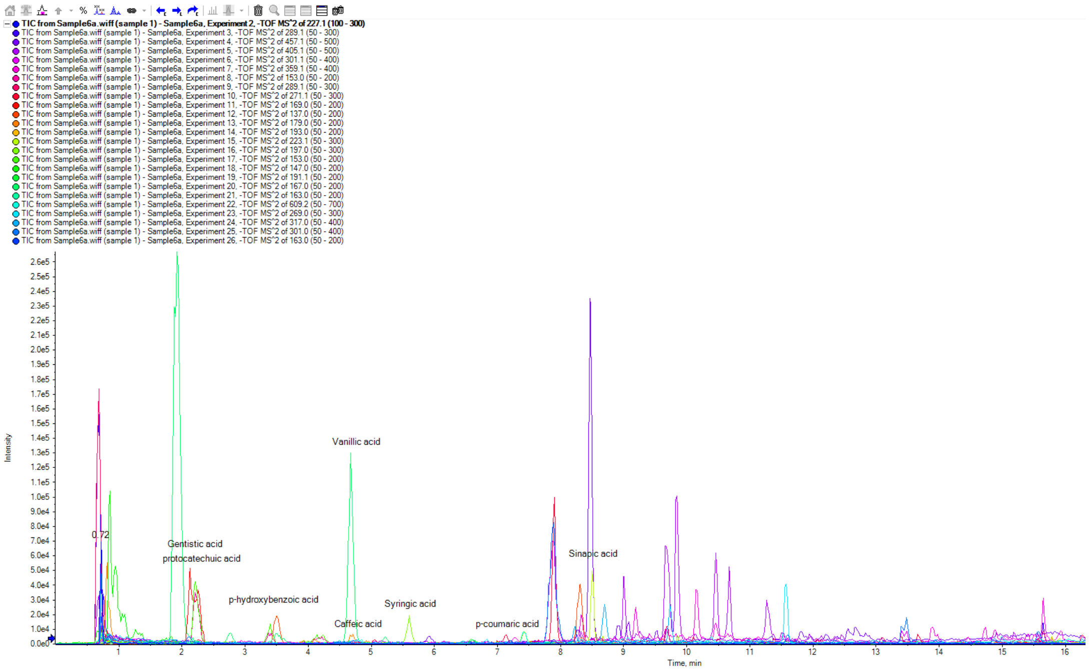

(**c**)

Figure S3. Chromatograms of *V. polita*, (**a**) methanolic extract, (**b**) ethanolic extract, (**c**) water extract.
